# Ultrasonographic Demonstration of Severe Right-Sided Colitis in Concurrent Enterohemorrhagic *Escherichia coli* O157 and *Campylobacter jejuni* Infection

**DOI:** 10.3390/diagnostics16183041

**Published:** 2026-09-19

**Authors:** Kousuke Kubota, Koji Hayashi, Yohei Midori

**Affiliations:** 1 Department of Gastroenterology, Fukui General Hospital, 55-16-1 Egami, Fukui 910-8561, Japanymidori@u-fukui.ac.jp (Y.M.); 2 Department of Rehabilitation Medicine, Fukui General Hospital, 55-16-1 Egami, Fukui 910-8561, Japan

**Keywords:** *Escherichia coli* O157, *Campylobacter jejuni*, coinfection, gastroenteritis, diagnosis, differential

## Abstract

A previously healthy 23-year-old woman presented with acute diarrhea, abdominal pain, and bloody mucoid stool following raw beef consumption, with a fever of 37.8 °C. On admission, her vital signs were unremarkable. Blood tests revealed elevated C-reactive protein, hypokalemia, and hypoproteinemia. Abdominal ultrasonography clearly demonstrated severe right-sided colitis characterized by marked circumferential bowel-wall thickening (~2 cm), loss of normal wall stratification, and continuous involvement from the ascending to proximal transverse colon. To avoid unnecessary radiation exposure in this young, clinically stable patient, computed tomography (CT) was omitted. Stool culture yielded enterohemorrhagic *Escherichia coli* (EHEC) O157 and *Campylobacter jejuni*. While ultrasonography effectively delineated the severity and continuous extent of colitis, imaging alone could not differentiate between these pathogens or identify the concurrent coinfection. Furthermore, this dual infection presented a major therapeutic challenge: guidelines suggest macrolides for severe *Campylobacter* enteritis, but antimicrobials are strictly discouraged in EHEC O157 infection due to the risk of hemolytic uremic syndrome (HUS). Managed conservatively with fluid replacement and probiotics without antimicrobial therapy, she recovered uneventfully and was discharged on hospital day 9. This case highlights the primary diagnostic utility and safety of ultrasonography in evaluating acute right-sided colitis and underscores the importance of cautious supportive care when navigating conflicting therapeutic guidelines in rare gastrointestinal coinfections.

**Figure 1:**
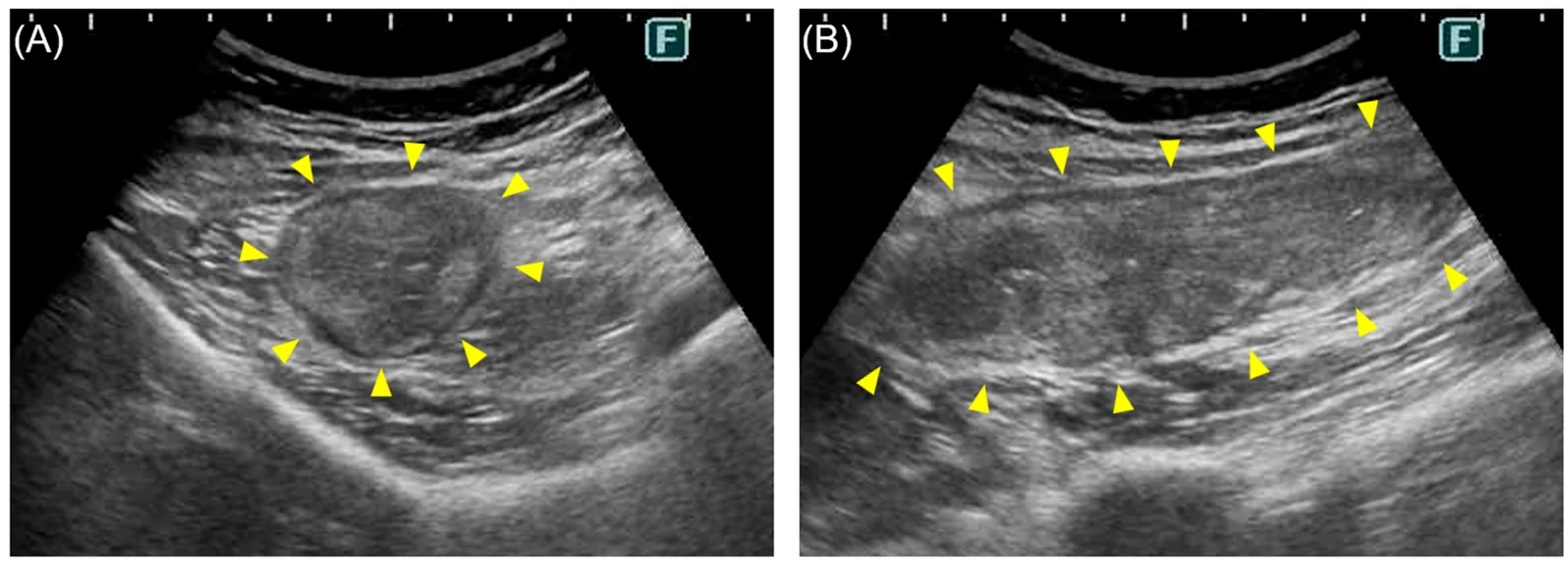
Abdominal ultrasonography on admission. (**A**) Short-axis and (**B**) long-axis views of the ascending colon showing marked circumferential bowel-wall thickening of approximately 2 cm (yellow arrowheads) with loss of normal bowel-wall stratification. Similar changes extended continuously from the ascending colon to the proximal transverse colon. This case represents a rare coinfection with EHEC O157 and *Campylobacter jejuni* following raw beef consumption. Confirmed coinfection with these pathogens is extremely rare; for instance, only one case was identified among 775 suspected patients during the 1999 New York waterborne outbreak [1]. In our patient, abdominal ultrasonography played a central diagnostic role by clearly demonstrating severe right-sided colitis and delineating the continuous extent of bowel involvement. Given her young age and clinical stability, abdominal computed tomography (CT) was omitted to avoid unnecessary radiation exposure, as ultrasonography provided sufficient diagnostic detail to guide immediate management. Although CT remains a valuable complementary tool when ultrasonographic visualization is limited by bowel gas or when surgical complications are suspected, neither ultrasonography nor CT can differentiate EHEC O157 from *Campylobacter jejuni*, nor can imaging identify the presence of a coinfection. This rare dual infection created a critical therapeutic challenge. Clinical guidelines consider antimicrobial therapy (e.g., macrolides) for severe *Campylobacter* enteritis presenting with severe abdominal pain and bloody stools; however, antimicrobial use in EHEC O157 infection is strictly discouraged due to the potential risk of triggering hemolytic uremic syndrome (HUS) [2]. Consequently, the identification of EHEC O157 dictated a conservative strategy, precluding targeted antibiotic treatment for *Campylobacter*. Although some studies in pediatric populations suggest that polymicrobial enteric infections may be associated with increased disease severity [3], our patient—a previously healthy young adult—achieved full recovery with supportive care and probiotics alone. This case highlights ultrasonography as an effective, non-invasive primary imaging modality for assessing the extent of acute right-sided colitis, and demonstrates that cautious supportive management can achieve favorable outcomes even when conflicting guidelines constrain antimicrobial choices.

## Data Availability

The original contributions presented in this study are included in the article. Further inquiries can be directed to the corresponding author.
